# The value of resting-state functional magnetic resonance imaging for detecting epileptogenic zones in patients with focal epilepsy

**DOI:** 10.1371/journal.pone.0172094

**Published:** 2017-02-15

**Authors:** Zhijuan Chen, Yang An, Bofeng Zhao, Weidong Yang, Qing Yu, Li Cai, Hongyan Ni, Jianzhong Yin

**Affiliations:** 1 Department of Neurosurgery, Tianjin Medical University General Hospital, Tianjin, China; 2 First Central Clinical College, Tianjin Medical University, Tianjin, China; 3 Radiology Department, Tianjin First Central Hospital, Tianjin, China; 4 Department of Neurology, Tianjin Medical University General Hospital, Tianjin, China; 5 Clinical PET-CT Center, Tianjin Medical University General Hospital, Tianjin, China; University of Texas at Austin, UNITED STATES

## Abstract

**Objective:**

To determine the value of resting-state functional magnetic resonance imaging (RS-fMRI) based on the local analysis methods regional homogeneity (ReHo), amplitude of low-frequency fluctuations (ALFF), and fractional ALFF (fALFF), for detecting epileptogenic zones (EZs).

**Methods:**

A total of 42 consecutive patients with focal epilepsy were enrolled. Sensitivity, specificity, positive predictive value (PPV), and negative predictive value (NPV) of visually assessed RS-fMRI, MRI, magnetic resonance spectroscopy (MRS), video electroencephalography (VEEG), and positron-emission tomography computed tomography (PET-CT) in EZ localization were evaluated to assess their diagnostic abilities. ReHo, ALFF, and fALFF were also compared for their diagnostic values.

**Results:**

RS-fMRI showed comparable sensitivity to PET (83.3%) and specificity to VEEG (66.7%), respectively, for EZ localization in patients with focal epilepsy. There were no significant differences between RS-fMRI and the other localization techniques in terms of sensitivity, specificity, PPV, and NPV. The sensitivities of ReHo, ALFF, and fALFF were 69.4%, 52.8%, and 38.9%, respectively, and for specificities of 66.7%, 83.3%, and 66.7%, respectively. There were no significant differences among ReHo, ALFF, and fALFF, except that ReHo was more sensitive than fALFF.

**Conclusions:**

RS-fMRI may be an efficient tool for detecting EZs in focal epilepsy patients.

## Introduction

Focal epilepsy accounts for approximately 60% of all epilepsy cases [1]. A selected subset of focal epilepsy patients may benefit from complete resection or total disconnection of the epileptogenic zone (EZ), which is defined as an area of the cortex indispensable for the generation of clinical seizures [2]. EZ localization of is crucial in the pre-operative evaluation for such patients. Although several standard techniques are available for EZ localization in the ictal or interictal state, all these modalities have inherent limitations. For example, EEG (including video EEG [VEEG] and intracranial EEG) and magnetoencephalography have the best temporal resolution, but poor spatial resolution and high cost greatly limit their application in EZ localization. Structural magnetic resonance imaging (MRI) has the best spatial resolution but is not sensitive enough to detect subtle structural lesions such as cortical dysplasia [3–4]. Magnetic resonance spectroscopy (MRS) can elucidate the neurochemical substrates of epilepsy and has important diagnostic value in patients with no overt morphological abnormalities but only metabolic and functional defects. However, MRS data interpretation may vary among researchers. Positron-emission tomography (PET) and single-photon emission computed tomography (SPECT) have improved the detection rate of inconspicuous lesions [5,6]. However, these methods expose the patient to radioactivity, and tend to reveal regional rather than local abnormalities. Furthermore, none of above methods is a substitute for the others. Although a combination of these techniques could improve the accuracy of localization, there is still a need for more precise, noninvasive techniques for preoperative EZ identification.

Recently, simultaneous EEG and functional MRI (fMRI), integrating high temporal and spatial resolution, has shown great promise in EZ localization [7,8]. In this method, simultaneous EEG provides time points of interictal epileptiform data, and subsequent event-related fMRI analysis is used to explore related changes in blood oxygen level—dependent (BOLD) signals, thereby greatly improving the accuracy of EZ localization [9]. However, practical issues such as high cost, complicated EEG data analysis, and time-consuming preparation limit the utility of this technique in clinical practice. Resting-state fMRI (RS-fMRI) [10] is another promising application of BOLD fMRI. Recent developments in data-driven analysis have enabled a widespread use of BOLD fMRI alone (i.e., without additional techniques such as EEG). Epilepsy is considered a typical disorder with abnormal spontaneous neuronal activity in the resting state [11], and several recent studies have shown that RS-fMRI may provide useful information for EZ localization [12–15]. Zhang firstly applied a RS-fMRI parameter, amplitude of low-frequency fluctuations (ALFF), in patients with mesial temporal lobe epilepsy (MTLE) and found a consistent distribution of increased ALFF in the mesial temporal lobe, as well as other cortical and subcortical structures; this was similar to the spatial pattern of the MTLE network proposed previously [12]. Similarly, regional homogeneity (ReHo), another fMRI parameter, has been successfully used to detect abnormal epileptic synchronization in pediatric patients with non-lesional temporal lobe epilepsy [13]. In addition, significantly higher ReHo in the bilateral thalamic region was observed in a group of patients with generalized tonic-clonic epilepsy [14]. Zeng et al. compared ReHo between patients with MTLE and hippocampus sclerosis (MTLE-HS) and healthy controls [15]. Interestingly, they found increased ReHo in almost the same area as that of increased ALFF reported by Zhang et al. [12], and proposed that increased ReHo in specific regions may form a network likely responsible for seizure genesis and propagation. The consistent results in the above studies suggest that these RS-fMRI parameters are suitable for clinical research. However, the above methods were used in epileptic patients with homogeneous etiologies, and their clinical values have not been thoroughly examined. Furthermore, neither a systematic comparison of RS-fMRI with other localization techniques nor the formal sensitivity and specificity assessment of these RS-fMRI parameters has been performed as yet. These investigations were undertaken in the present study. To this purpose, we used a comprehensive evaluation-defined EZ as a reference standard to calculate the sensitivity, specificity, positive predictive value (PPV), and negative predictive value (NPV) of RS-fMRI for EZ localization in a large and heterogeneous group of patients with focal epilepsy.

## Materials and methods

### Patients

We enrolled 42 consecutive patients (18 females and 24 males; mean age, 24 years; range, 5–50 years) with focal epilepsy, who underwent a comprehensive preoperative evaluation, including clinical examination, VEEG, PET-CT, high-resolution MRI epilepsy protocol, and RS-fMRI between September 2010 and January 2013. The diagnosis of focal epilepsy was established based on the International League Against Epilepsy recommendations [16]. An interdisciplinary epilepsy patient-management team consisting of specialists from the neurology, neurosurgery, nuclear medicine, and radiology departments attempted to identify a comprehensive evaluation-defined EZ in each individual. Patients with conflicting EZ localizations among imaging methods were excluded. Other exclusion criteria included epilepsy related to tumors and cerebral arteriovenous malformations, as well as traumatic and postoperative epilepsy. Subjects with claustrophobia, not cooperating were also excluded from this study.

Written informed consent was obtained from all subjects or parents for those under the age of 18 years. This research was approved by the institutional review board and the medical ethics committee of Tianjin First Central Hospital and all methods were performed in accordance with the relevant guidelines and regulations.

### VEEG

VEEG was recorded on an EASY-III system with 150 channels (Cadwell, Kennewick, Washington, USA). Closely spaced scalp electrodes were placed according to the international 10–20 system. The changes in EEG findings and behavior were monitored for at least 24 hours; if a typical ictal EEG not was recorded, the time might be prolonged to 48 hours. 34/42(81%) of the subjects had a typical ictal EEG. All interictal and ictal EEG findings were analyzed with VEEG recordings.

### MRI

MRI, MRS, and RS-fMRI were performed on a 3.0T Magnetom Trio instrument (Siemens, Erlangen, Germany) with a standard 32-channel head coil.

The epilepsy MRI protocol consisted of routine MRI and high-resolution coronal hippocampus anatomical images. For the latter, the hippocampus was displayed along and perpendicular to its longitudinal axis. Coronal sequences were obtained as follows: (i) T1-weighted inversion recovery sequence, repetition time (TR), 2510ms; echo time (TE), 9.6ms; inversion time (TI), 1038ms; field of view, 180mm; slice thickness, 3mm; interslice gap, 0.3mm; flip angle, 130°; matrix, 256×256; number of excitations, 2; (ii) T2-weighted turbo spin-echo sequence, same acquisition parameters as above, except that TR and TE was 5000ms and 93ms, respectively; and (iii) coronal fluid-attenuated inversion recovery sequence, TR, 9000ms; TE, 93ms; TI, 2500ms; slice thickness, 3mm; gap, 0.3mm. To detect subtle signal abnormalities and obtain a good gray matter contrast, we performed a specific axial inversion recovery sequence with the following parameters: TR, 1500ms; TE, 13ms; TI, 499 ms; slice thickness, 4mm; gap, 0.6mm; and flip angle, 150°.

### MRS

Bilaterally hippocampus was selected as default ROI on single-voxel ^1^H MRS (point-resolved spectroscopy sequence: TR, 2,000 ms; TE, 135 ms, with 128 and 96 acquisitions in the hippocampus and neocortex, respectively). If an abnormal was found in the neocortex during the MRI scan, the ROI of MRS would be moved to the lesion and symmetrical areas of the contra lateral hemisphere. The volume of interest was represented by a rectangle in the hippocampus and a square in the neocortex, and included the maximal target area in the axial, sagittal, and coronal orientations; its size was based on the lesion observed by MRI. The concentrations of choline compounds (Cho), total creatine (Cr), and *N*-acetylaspartate (NAA) were automatically obtained, and signal ratios of Cho/(Cr+Cho) and NAA/(Cr+Cho) were calculated in the hippocampus and neocortex. A bilateral difference over 15% was considered significant.

### RS-fMRI

RS-fMRI was performed with continuous data acquisition (T2-weighted single-shot echo planar imaging sequence: field of view, 220mm; matrix, 256×256; slice thickness, 5mm; TR, 2000 ms; TE, 30ms; 300 frames) for 20min, followed by a three-dimensional T1-weighted anatomical sequence (field of view, 220mm; matrix, 256×256; 176 sagittal slices; slice thickness, 4mm; TR, 400ms; TE, 8.9ms; flip angle, 70°) for coregistration with functional images. The first 10 functional images were discarded to ensure steady-state tissue magnetization. To ensure precise EZ localization, the data were preprocessed and analyzed with SPM8 (Wellcome Institute of Cognitive Neuroscience, London, UK) and REST (Resting-State fMRI Data Analysis Toolkit V1.8, Beijing, China), respectively, to generate activation maps. The former involved the following steps: rigid-body realignment to correct for subject motion, coregistration, spatial normalization with a resampling voxel size of 3×3×3 mm^3^, spatial smoothing with an 8×8×12.5 mm full width at half maximum (FWHM) Gaussian kernel. The patients whose head motion exceeded 1.0mm or involved a rotation exceeding 1.0° during the fMRI scanning were excluded. After data preprocessing with SPM, we processed the RS-fMRI for ReHo, ALFF, and fALFF with the REST Toolkit [17], examined different threshold level maps, and compared bilateral brain hemisphere and hippocampus to identify the possible EZ by visual analysis. If one or more of the three RS-fMRI indexes were concordant with the final comprehensive evaluation, the results of given index and those of RS-fMRI were considered to be positive; otherwise, the results were considered negative.

### PET/CT

Imaging was performed on an integrated PET-CT system (Discovery LS, GE Medical Systems, Milwaukee, USA) after intravenous injection of 222-370MBq (6-10mCi) ^18^F-fluorodeoxyglucose (FDG) 60 min before scanning. All patients were fasted for at least 6 hours before the PET/CT study. A three-dimensional scan mode was applied with an 8-min acquisition per bed position. The intrinsic spatial resolution of PET was 6.0mm (FWHM) in the center of the field of view. Transmission CT and emission PET images were reconstructed using a default vendor-implemented iterative reconstruction algorithm. All images were acquired without breath-holding. Attenuation-corrected PET and CT images were reconstructed in the axial, coronal, and sagittal planes, with a slice thickness of 5 mm, and merged using a workstation.

### Image interpretation and analysis

Localization results, which were limited to the sublobar level at least, by VEEG, MR studies (MRI, MRS, and RS-fMRI), and PET/CT, were interpreted independently by a neurologist (Q.Y., 20 years of experience), a neurosurgeon(W. Y., with 15 years of experience), an electrical specialists, (Z. C., with 8 years of experience), a neuroradiologist (J. Y., with 15 years of experience), and a nuclear medicine physician (L. C., with 15 years of experience in PET/CT). They were all informed about the patient-specific clinical background (e.g., clinical features); for an independently interpretation, the results of other methods were blinded.

### Reference standard

After the independent interpretation, our interdisciplinary epilepsy patient—management team consisting of specialists from the neurology, neurosurgery, nuclear medicine, and radiology departments discussed the data for each patient, attempted to carry out a comprehensive evaluation, and reach an agreement for the localization which would serve as the reference standard.

### Statistical analysis

The sensitivity, specificity, PPV, NPV, and Youden index (YI) of each imaging modality and the three RS-fMRI indexes for EZ localization were assessed in patients with focal epilepsy. The significance of differences in sensitivity, specificity, PPV, and NPV was evaluated using the chi-square test or where appropriate, the Yates correlation and Fisher exact tests. A commercially available software (SPSS version 17.0; SPSS Inc., Chicago, IL, USA) was used for all statistical analyses. The differences were considered statistically significant at p<0.05. Since multiple comparisons were made among the three indexes, the differences were considered statistically significant at corrected p<0.0125.

## Results

### Types of focal epilepsy

An EZ was successfully identified in 36 (85.7%) patients (18 females and 18 males). No EZ was found in the remaining 6 (14.3%) male patients who were diagnosed with idiopathic focal epilepsy (IFE). The types of epilepsy diagnosed base on the comprehensive evaluation are presented in Table 1. The focal epilepsy types diagnosed were divided into MTLE and neocortical epilepsy. Patients with neocortical epilepsy included 9 malformation of cortical development (MCD), which 4 had a surgery with a pathologic result and 5 showed overt MRI manifestations. The epileptogenic zone was defined at the temporal cortex in the remaining 5 neocortical epilepsy cases, but these subjects declined surgery and had no characteristic appearance on imaging.

**Table 1:** Types of focal epilepsy.

Types	N (%)	Males/ Females	Age	Surgery
MTLE	22 (52.4%)	10/12	27.8±11.6	5
NE				
MCD	9 (21.4%)	5/4	21.1±11.6	4
Others	5 (11.9%)	3/2	25.8±9.7	
IFE	6 (14.3%)	6/0	17.0±7.2	
Total	42(100%)	24/18	24.6±11.3	9

Abbreviations: MTLE, mesial temporal lobe epilepsy; NE, neocortical epilepsy; MCD, malformation of cortical development; IFE, idiopathic focal epilepsy.

Among the 22 MTL epilepsy cases, 5 underwent surgery. Seizure was controlled in all 5 cases, but 3 still had medicines during 3 year follow-up after surgery. Among the 9 MCD subjects, 4 underwent surgery, 3 were seizure-free within 3 years after the operation. One case with extensively large MCD still had seizure after the operation.

### Typical cases

**Fig 1:**
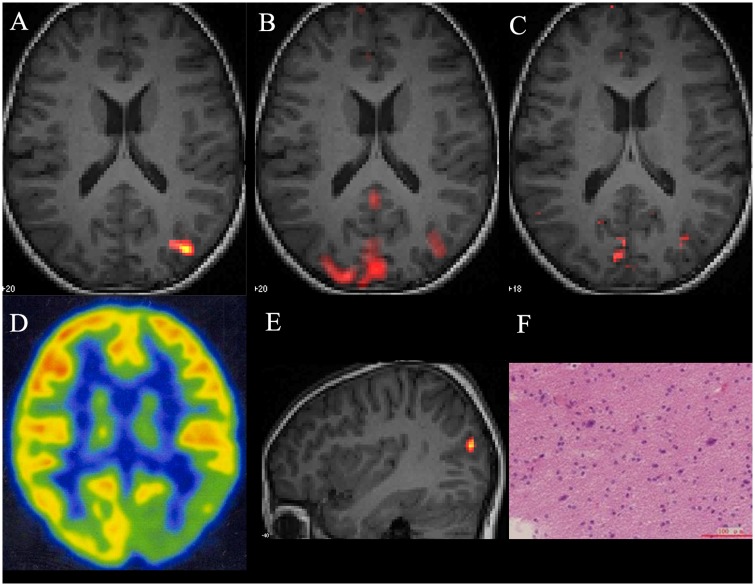
Left parieto-occipital lobe dysplasia in a MRI negative 9-year-old patient. (A) Axial and (E) sagittal fALFF, (B) axial ALFF, and (C) ReHo reveal abnormal activation in the left parieto-occipital lobe. (D) The corresponding ^18^F-FDG PET/CT image shows low FDG uptake in the left frontoparietal region. (F) Pathological confirmation of left parieto-occipital lobe focal cortical dysplasia.

**Fig 2:**
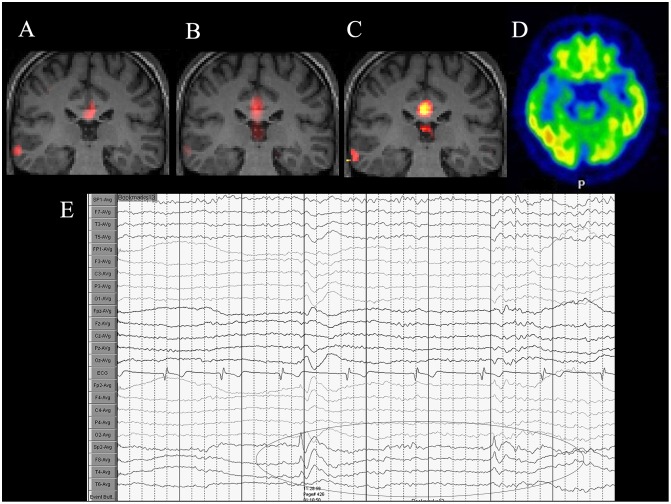
A 38-year-old man diagnosed with right lateral temporal lobe epilepsy. (A) Coronal fALFF, (B) ALFF, and (C) ReHo reveal abnormal activation in the right lateral temporal lobe. An area of activation could also be found in the corpus callosum, which is considered a part of the default mode network. (D) The corresponding ^18^F-FDG PET/CT image reveals low FDG uptake in the right lateral temporal lobe region. (E) VEEG depiction of interictal epileptiform discharges of right temporal lobe.

**Fig 3:**
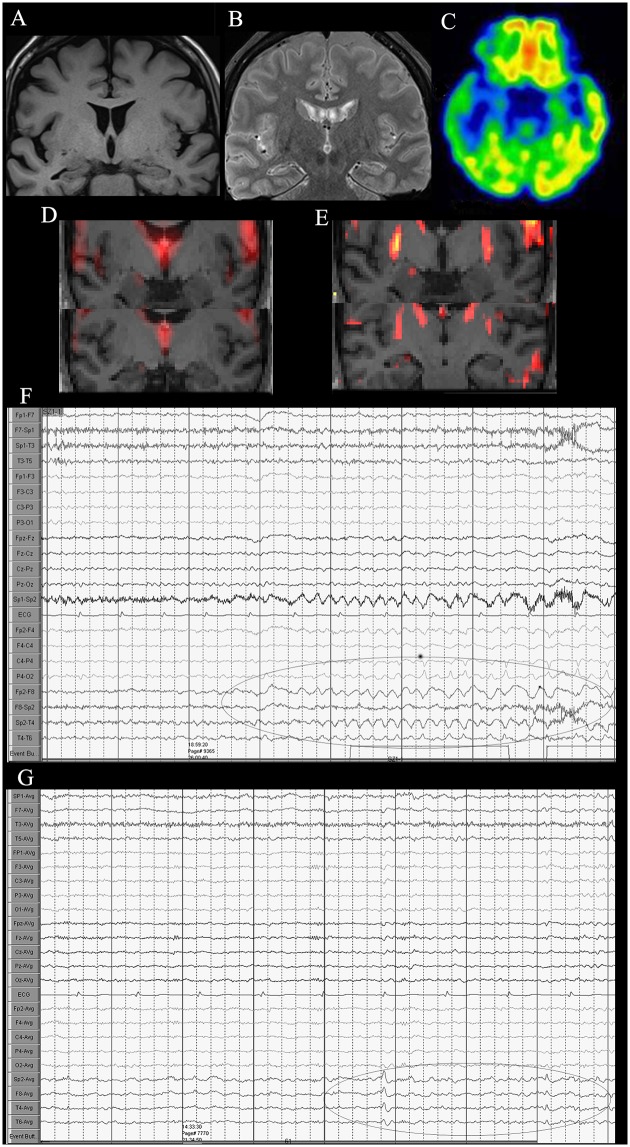
A 42-year-old woman diagnosed with right mesial temporal lobe epilepsy. (A) Coronal T1-weighted image showing right hippocampus atrophy, (B) FLAIR image showing slightly high signal of right hippocampus. (C) ^18^F-FDG PET/CT imaging reveals low FDG uptake in the left temporal lobe region, particularly, in the hippocampus. (D) Coronal ALFF and (E) ReHo reveal abnormal activation in the right hippocampus compared to the left side. (F, G) VEEG showing ictal and interictal discharges in the right temporal lobe.

Figs 1 and 2 depict two typical cases of neocortical epilepsy. The first was a focal cortical dysplasia with negative MRI, which was confirmed by surgery. In the second case, epileptogenic zone was defined at the right lateral temporal cortex, but the patients declined the surgery finally. Fig 3 depicts a case with right mesial temporal lobe epilepsy.

### Diagnostic values of various techniques

Table 2 summarizes the results of MRI, MRS, VEEG, FDG-PET, and RS-fMRI, with the comprehensive evaluation-defined EZ as the reference standard. MRI correctly identified the EZ in 50% of all patients (14/22 with MTLE; 7/14 with NE). There were 2 MRI negative MCD patients, which lesion was detected on PET and RS-fMRI (Fig 1). MRS showed abnormalities in 42.9% of all patients (12/22 with MTLE; 6/14 with NE). VEEG correctly captured abnormal EZ spikes in 76.2% of all patients (19/22 with MTLE; 13/14 with NE). On FDG-PET, hypometabolism was correctly identified in 71.4% of all patients (17/22 with MTLE; 13/14 with NE). RS-fMRI also correctly identified the EZ in 71.4% of all patients (19/22 with MTLE; 11/14 with NE).

**Table 2:** Performance of various imaging techniques for EZ localization.

	TP	FN	FP	TN	Sensitivity (%)	Specificity (%)	PPV (%)	NPV (%)	YI
VEEG	32	4	2	4	88.9	66.7	94.1	50.0	0.57
MRI	21	15	1	5	58.3	83.3	95.5	25.0	0.42
MRS	18	18	0	6	50.0	100	100	25.0	0.50
PET-CT	30	6	3	3	83.3	50.0	90.9	33.3	0.33
RS-fMRI	30	6	2	4	83.3	66.7	93.8	40.0	0.50

EZ, epileptogenic zone; TP, true positive; FN, false negative; FP, false positive; TN, true negative; PPV, positive predictive value; NPV, negative predictive value; YI, Youden index; VEEG, video electroencephalography; MRI, magnetic resonance imaging; MRS, magnetic resonance spectroscopy; PET-CT, positron emission tomography—computed tomography; RS-fMRI, resting- state functional MRI.

The sensitivity, specificity, PPV, NPV, and YI for each diagnostic modality are shown in Table 2. VEEG had the highest sensitivity (88.9%). The sensitivities of FDG-PET and RS-fMRI (83.3% for both) were lower than that of VEEG but higher than values for MRI (58.3%) and MRS (50.0%). MRS had the highest specificity (100%) and PPV (100%), followed by MRI (83.3% and 95.5%, respectively), VEEG (66.7% and 94.1%, respectively), RS-fMRI (66.7% and 93.8%, respectively), and FDG-PET (50% and 90.9%, respectively). VEEG had a higher NPV (50.0%) compared with RS-fMRI (40.0%), FDG-PET (33.3%), MRI (25.0%), and MRS (25.0%). Table 3 summarizes statistical differences between RS-fMRI and the other localization techniques in terms of sensitivity, specificity, PPV, and NPV. Sensitivity of RS-fMRI was significantly higher than that of MRI (χ^2^ = 5.45, P = 0.0196) and MRS (χ^2^ = 9.00, P = 0.0027), while its specificity, PPV, and NPV did not significantly differ from MRI and MRS values. The sensitivity, specificity, PPV, and NPV of VEEG and FDG-PET did not differ from those of RS-fMRI.

**Table 3:** Comparison of RS-fMRI with other EZ localization techniques.

	Sensitivity		Specificity		PPV		NPV	
	χ^2^	P	χ^2^	P	χ^2^	P	χ^2^	P
MRI	5.45	0.0196	/	1.0000	0.57	0.4500	/	0.4311
MRS	9.00	0.0027	/	0.4545	/	0.5298	/	0.4309
VEEG	0.46	0.4955	/	1.0000	0.21	0.6501	/	1.0000
FDG-PET	0	1.0000	/	1.0000	0	0.2100	/	1.0000

RS-fMRI, resting-state functional magnetic resonance imaging; EZ, epileptogenic zone; PPV, positive predictive value; NPV, negative predictive value; MRI, magnetic resonance imaging; MRS, magnetic resonance spectroscopy; VEEG, video electroencephalography; FDG-PET, fluorodeoxyglucose positron emission tomography.

### Diagnostic values of the three RS-fMRI indexes

Tables 4 and 5 summarize the EZ localization results for ReHo, ALFF, and fALFF. As shown in these tables, ReHo was superior or identical to ALFF and fALFF in the accuracy for EZ localization.

**Table 4:** EZ localization rates of ReHo, ALFF, and fALFF.

	ReHo	ALFF	fALFF
MTLE	15/22	10/22	4/22
NE	10/14	9/14	10/41
IFE	2/6	0/6	2/6
Total	27/42	19/42	16/42

ReHo, regional homogeneity; ALFF, amplitude of low-frequency fluctuations; fALFF, fractional ALFF; MTLE, mesial temporal lobe epilepsy; NE, neocortical epilepsy; IFE, idiopathic focal epilepsy.

**Table 5:** Performance of ReHo, ALFF, and fALFF for EZ localization.

	TP	FN	FP	TN	Sensitivity(%)	Specificity(%)	PPV(%)	NPV(%)	YI
ReHo	25	11	2	4	69.4	66.7	92.6	26.7	0.36
ALFF	19	17	1	5	52.8	83.3	95.0	22.7	0.36
fALFF	14	22	2	4	38.9	66.7	87.5	15.4	0.06

ReHo, regional homogeneity; ALFF, amplitude of low-frequency fluctuations; fALFF, fractional ALFF; EZ, epileptogenic zone; TP, true positive; FN, false negative; FP, false positive; TN, true negative; PPV, positive predictive value; NPV, negative predictive value; YI, Youden index.

Table 5 summarizes the diagnostic values of ReHo, ALFF, and fALFF based on the comprehensive evaluation. ReHo had higher sensitivity (69.4%) and NPV (26.7%) than ALFF (52.8% and 22.7%, respectively) and fALFF (38.9% and 15.4%, respectively); ALFF had higher specificity (83.3%) and PPV (95.0%) than ReHo (66.7% and 92.6%, respectively) and fALFF (66.7% and 87.5%, respectively). ReHo and ALFF had the same YI (0.36), which was superior to that of fALFF (0.06).

Table 6 summarizes the statistical differences among the three RS-fMRI indexes in terms of sensitivity, specificity, PPV, and NPV. No significant differences were observed between ReHo and ALFF on one hand, and between ALFF and fALFF on the other hand, in terms of sensitivity and specificity. Specificity also did not significantly differ between ReHo and fALFF (Fisher exact test, P = 1.0000); only sensitivity was higher with ReHo than fALFF (χ^2^ = 6.77, p = 0.0093).

**Table 6:** Comparison of the three RS-fMRI indexes.

	Sensitivity		Specificity		PPV		NPV	
	χ^2^	P	χ^2^	P	χ^2^	P	χ^2^	P
ReHo vs. ALFF	2.10	0.1496	/	1^b^	0.07	0.7875^a^	/	1.0000^b^
ReHo vs. fALFF	6.77	0.0093	/	1^b^	0	0.9899^a^	0.22	0.6391^a^
ALFF vs. fALFF	1.40	0.2370	/	1^b^	/	0.5742^b^	0.08	0.7808^a^

RS-fMRI, resting-state functional magnetic resonance imaging; PPV, positive predictive value; NPV, negative predictive value; ReHo, regional homogeneity; ALFF, amplitude of low-frequency fluctuations; fALFF, fractional ALFF.

^a^ Yates correlation,

^b^ Fisher exact test

## Discussion

### Epilepsy and RS-fMRI indexes

Epilepsy is caused by abnormal neuronal discharges. And seizure is inherently a network event, involving numerous neurons firing synchronously [18]. The epileptogenic properties of neuronal tissues are determined by two hallmarks: neuronal hyperexcitability and hypersynchrony of local or widespread neuronal networks of hippocampal and cortical neurons [18, 19]. While these two sets of changes facilitated the structural and functional delineation of epileptogenic regions [20], the advent of new fMRI post-processing methods has enabled the investigation of previously overlooked aspects of intrinsic (abnormal) spontaneous brain activity [21]. Among these methods, we focused on two local voxel-based measures within three analytic methods: ReHo [22], ALFF [23], and fALFF [24]. The ReHo analysis is a more stable voxel-based measure—rather than a linear statistical measure—that is based on the theory according to which similarity in BOLD-signal fluctuations in a local brain region reflects the homogeneity of neuronal activity at the same frequency. ReHo is thought to reflect the extent of temporal homogeneity and synchrony changes in neuronal activity. ALFF is defined as the total power within a defined low frequency range (i.e., 0.01–0.1 Hz), and calculated using the voxel-wise magnitude of specific frequency bands. Since there are coherent low-frequency fluctuations in BOLD signals in widespread but functionally related brain regions, the ALFF can indicate both the nature and extent of signal changes underlying spontaneous neuronal activities. Although ALFF effectively detects low frequency fluctuations, the fluctuations detected can extend over 0.1Hz, particularly near major vessels, which are characterized by widespread oscillations across both low and high frequencies [25]. To reduce sensitivity to physiological noise, Zou et al. proposed the fALFF parameter [24], which can be regarded as a normalized ALFF at the individual level. The fALFF is calculated by dividing the ALFF by total energy over the detectable frequency range. Both ALFF and fALFF reflect the magnitude of spontaneous neuronal activity [22, 23]. Increased ALFF and ReHo was described in the hippocampus and adjacent structures in MTLE patients [12, 13, 15]. The hippocampus plays an important role in MTLE, and is rarely detectable using BOLD fMRI in normal subjects [26, 27]. Additionally, increased ReHo in the thalamus has been found in patients with generalized tonic-clonic seizures [14]. The above results indicate that hippocampal and thalamocortical circuit abnormalities are the underlying pathophysiological substrate of MTLE and generalized tonic-clonic seizures, respectively. The enhanced coherent neuronal activity and fluctuation amplitude within spatially organized brain regions were thought to reflect BOLD signal activation induced by the epileptic activity [12, 13]. Thus, from a simplistic perspective, ReHo can be used as ideal parameter of temporal relevance, while ALFF and fALFF reflect the intensity of regional (abnormal) neuronal activities in the brain. Thus, RS-fMRI can constitute an ideal candidate for studying metabolic changes induced by abnormal spontaneous neuronal activities in the interictal period.

### Value of RS-fMRI in various types of focal epilepsy

As stated above, previous RS-fMRI studies have focused on patients with homogeneous etiologies of epilepsy [12–15]. In this study, we used a comprehensive evaluation-defined EZ based on multimodal tests as a reference standard to determine the usefulness of various EZ localization methods in a large cohort of patients with different types of focal epilepsy.

#### MTLE

In MTLE patients, the sensitivity of RS-fMRI was 86.4% (19/22). Most activation areas were in the hippocampal region, composed of the hippocampus, amygdala, and the parahippocampal region, which is consistent with previous reports [12,13,15]. These findings provide further evidence for hippocampus involvement in the generation of interictal or ictal discharges.

#### Neocortical epilepsy

In neocortical epilepsy patients, PET had a good sensitivity (92.9%, 13/14) in identifying cortical lesions. RS-fMRI localized the EZ in 11 patients (78.6%), and showed higher sensitivity than MRI (50%, 7/14) and MRS (42.9%, 6/14). Although VEEG (92.9%, 13/14) had the same sensitivity as PET, it should be noted that the spatial resolution of EEG was still lower than other imaging techniques.

Among the 36 patients in whom an EZ was localized, MRI alone only showed positive findings in 21 patients (sensitivity 58%). In comparison, RS-fMRI alone identified the EZ in 71.4% of patients. combined MRI and RS-fMRI would help detect the EZ in 91.7% (33/36) of patients. Thus, our results suggest that RS-fMRI is useful in MRI negative patients (Fig 1) in whom the lesion was not detected on structural MRI but observed on RS-fMRI; this was subsequently confirmed by PET and histology. The combination of MRI and RS-fMRI is easy in clinic; it would be interesting for future studies to confirm whether such combination increases the EZ detection rate. RS-fMRI showed promise in detecting subtle and MRI negative cases.

In the current analysis, the concordance between local activation and metabolic abnormalities (e.g., PET), both of which have been demonstrated to be linked to interictal epileptiform discharges (IEDs), suggested a potential relationship between local BOLD signal changes and spikes. In several patients, MRI (2 with MTLE, 1 with MCD) and PET (3 with MTLE, 3 with NE) showed typical positive results, but no obvious relevant findings were obtained by RS-fMRI. Factors that may have affected the sensitivity of RS-fMRI in these cases include the following: (1) RS-fMRI relies on the recording of IEDs during the scanning period. If few IEDs occur during fMRI data acquisition, the likelihood of detecting changes in BOLD signals decreases considerably. Since it is not possible to predict when epileptic discharges occur, the acquisition period may include no or few discharges. (2) Neurotransmitter levels may also influence the changes in BOLD signals [28]. In this study, some subjects remained on their regular medications before RS-fMRI scans. Select antiepileptic drugs may suppress IEDs in some epilepsy syndromes by altering the coupling between metabolism and function. In addition, a young child (with MTLE, 5 years old) required sedation during data collection. Sedation and antiepileptic medication may have influenced the results observed in the current study. It was shown that sedation affects the amplitude of low-frequency oscillations [29] and alters the BOLD signal [30]. Another limitation of this study is that only 9/42 patients had a surgery and pathological results; future studies including intracranial EEG and surgical outcome data should further evaluate the clinical value of RS-fMRI in patients with medically refractory focal epilepsy.

## Conclusions

The objective of this pilot study was to determine the value of RS-fMRI based on ReHo, ALFF, and fALFF in patients with focal epilepsy in an attempt to understand the potential of this technique to provide important and unique information regarding EZ localization. The sensitivity of RS-fMRI was comparable to that of FDG-PET, and its specificity similar to that of VEEG. Although the translation to routine clinical use is still distant, the present findings suggest that RS-fMRI, which has the advantages of non-invasiveness, fairly high temporal and spatial resolution, and easy implementation, could be a powerful and efficient tool for further clinical application.

## Supporting information

S1 File One case followup that still has seizure after operation. (DOC)

S1 Movie Review of RS-fMRI maps. (MP4)
