# Supplementary material for: The value of resting-state functional magnetic resonance imaging for detecting epileptogenic zones in patients with focal epilepsy
Source: PLoS One. 2017 Feb 15;12(2):e0172094. doi: 10.1371/journal.pone.0172094 (PMC5310782; doi:10.1371/journal.pone.0172094)
Supplement: S1 File — (DOC) [file pone.0172094.s001.doc]

**S1 File. One case followup that still has seizure after operation.**

This case had an extensive large MCD in the left temporal lobe, and the MCD tissue could not be totally removed (Figure below). The patient was a young lady and wanted to get pregnant. So the surgery was decided to remove MCD tissue at front temple lobe, and fulgerize the left hippocampus, hoping this could break the pathway of seizure, but failed to get a good result.


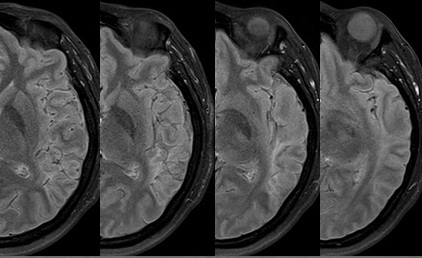


This was the cases involved at the very beginning of our works, and we did not get a clear idea of RS-fMRI at that time, especially before operation. The resting state fMRI was scanned both before and after the operation.

The RS-fMRI before operation: an activation area could be found at the up part of left temporal lobe (figure below).


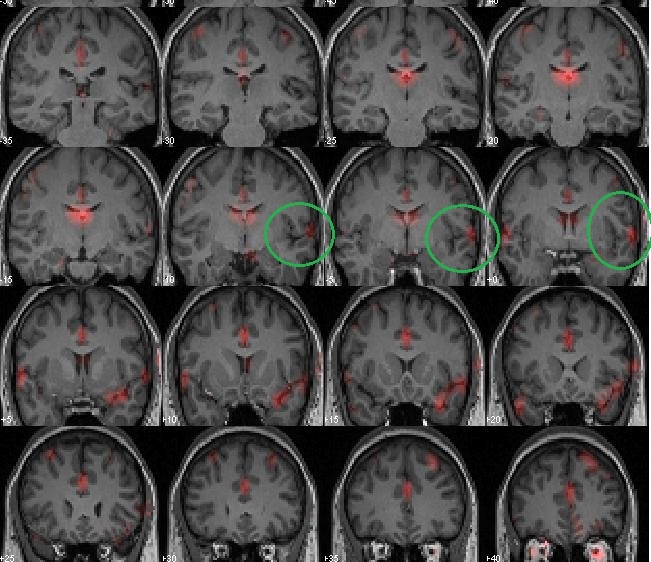


The RS-fMRI after operation: we still can find the activation in the same area (figure below).


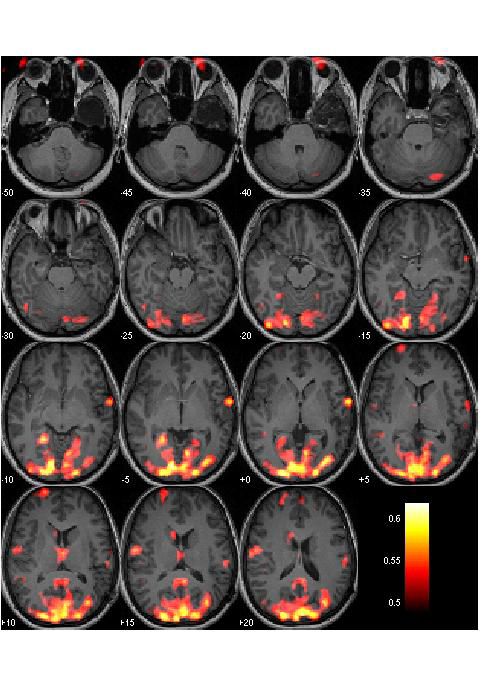


After this case, we begin to realize the value of RS-fMRI in the pre-operation evaluation of epilepsy. Despite the lesion was clear, but no any other method can reach the space resolution of RS-fMRI, compare to EEG and PET, which is extremely important for the operation plan.
